# The co-occurrence of the two main oral diseases: periodontitis and dental caries

**DOI:** 10.1007/s00784-023-05253-2

**Published:** 2023-09-16

**Authors:** Giacomo Baima, Hye-Sun Shin, Mariantonietta Arrica, Andreina Laforí, Massimo Cordaro, Mario Romandini

**Affiliations:** 1 Department of Surgical Sciences, C.I.R. Dental School, University of Turin, Turin, Italy; 2 Department of Dental Hygiene, Dongnam Health University, Suwon-Si, Gyeonggi-Do Korea; 3 Department of Medical, Surgical and Experimental Sciences, School of Dentistry, University of Sassari, Sassari, Italy; 4 Division of Fixed Prosthodontics and Biomaterials, Clinic of Dental Medicine, University of Geneva, Geneva, Switzerland; 5 Institute of Dentistry and Maxillofacial Surgery, Fondazione Policlinico Universitario A. Gemelli IRCCS, Catholic University of the Sacred Heart, Rome, Italy; 6 Department of Periodontology, Faculty of Dentistry, University of Oslo, 69, 0455 Geitmyrsveien, Oslo Norway

**Keywords:** Periodontal diseases, Dental caries, Risk factors, Epidemiology

## Abstract

**Objectives:**

Limited studies are available testing through multiple regression models the association between the two main oral diseases: dental caries and periodontitis. The aim of this cross-sectional population-based study was to verify whether dental caries and periodontitis co-occur in a representative sample of the South Korea population.

**Materials and methods:**

A total of 23,405 subjects representative of 36.2 million of adults (KNHANES) were examined. Univariate and multiple regression analyses using 7 different models were applied, controlling for age, gender, smoking status, frequency of toothbrushing, use of interproximal toothbrushes and flossing, educational level, income, gum diseases treatment and tooth filling in the previous year, BMI, Vitamin D serum levels, alcoholism, diabetes status, stress and carbohydrates dietary intake.

**Results:**

In the fully adjusted model, participants with periodontitis had, respectively, a mean of 0.82 (95% CI: 0.41–1.23) and of 0.36 (95% CI: 0.22–0.50) more untreated decayed surfaces and teeth than participants without periodontitis, with an OR to have at least one untreated decayed surface of 1.96 (95% CI: 1.66–2.32). However, cumulative caries experience (DF scores) and periodontitis were not associated.

**Conclusions:**

In this large nationally representative population, periodontitis and untreated dental caries co-occur. However, when considering cumulative caries experience (DF scores), the two diseases do not appear related.

**Clinical relevance:**

In light of their possible co-occurrence, clinicians should implement integrative diagnostic, preventive and treatment strategies for both diseases.

**Supplementary Information:**

The online version contains supplementary material available at 10.1007/s00784-023-05253-2.

## Introduction

Taken together, periodontitis and dental caries are among the most common diseases of humans and the leading cause of tooth loss [1–3]. They can lead to negative impacts upon quality of life, general health, and they pose a major burden on public health systems [4–10].

Although they exhibit a distinct pathophysiology, the emergence of a bacterial dysbiosis in genetically or environmentally susceptible individuals is the main etiologic factor for both these chronic complex diseases. Other shared determinants of susceptibility have been identified, with oral hygiene, nutrition, social, educational and economic factors providing the highest level of evidence. A plausible link between caries and periodontitis could therefore be hypothesized.

In 2017, the European Federation of Periodontology (EFP) and the European Organisation for Caries Research (ORCA) joined in the 1st European Workshop on Periodontal diseases and Dental Caries with the aim of assessing the interdependency between the two conditions and providing behavioural guidelines to clinicians, researchers and health policy-makers [11–19]. In the consensus report of Group 3, a paucity of studies analysing the co-occurrence of dental caries and periodontitis was highlighted [13]. Indeed, only few studies have been published on this topic showing contradictory results, which suggested either a direct association [20], an inverse association [21] or no association [22]. The most robust recent data reported a direct association [23, 24], but limited attempts were made to study whether this association is independent or entirely explainable by the common risk factors, i.e. age, socioeconomic level or access to dental care [25].

Having clear information on the epidemiological relationship between periodontitis and dental caries would result to uttermost importance in order to set individual and population-based prevention strategies, as well as to correctly plan comprehensive oral treatments. Therefore, this cross-sectional population-based study aimed to verify whether dental caries and periodontitis co-occur, and also, in case an association is found, if common risk factors can explain it.

## Materials and methods

This nationally representative cross-sectional study was reported according to the STrengthening the Reporting of OBservational studies in Epidemiology (STROBE) guidelines [26, 27].

### Study sample: KNHANES V and VI

Data for this study were obtained from the 2010 and 2012 sections of the Fifth Korea National Health And Nutrition Examination Survey (KNHANES V) and from the 2013–2015 sections of KNHANES VI. The 2011 section of KNHANES V was excluded as it didn’t pass the quality control for periodontal evaluation.

KNHANES is a nationwide cross-sectional survey promoted by the Korea Centre for Disease Control and Prevention (KCDC) and conducted on an annually representative sample of the total non-institutionalized South Korea population [28]. Written informed consent was obtained from all participants.

#### Sampling methods

KNHANES applied a clustered stratified multistage sampling protocol with a rolling survey model.

Each survey initially selected 192 Primary Sampling Units (PSUs) from around 200,000 geographically defined areas in the whole of South Korea. For each targeted PSU, 20 targeted households were chosen through systematic sampling, so that 3840 households were selected. A PSU consisted of approximately 50–60 households. The surveys included all subjects living in the enrolled households, who were at least 1 year old.

Due to statistical purposes, sample weights were set for sample participants. This way the whole South Korea population was represented and the complex survey design, the null response and the post-stratification (performed either by sex and age) were considered.

#### Survey contents

KNHANES survey consisted of three parts: the health interview, the health examination and the nutrition survey. Further information about the study design and methods are reported elsewhere [28].

### Assessment of study variables

A total of 25–30 trained and calibrated public health dentists assessed each year the dental and periodontal status of the participants according to the WHO criteria for oral health surveys [29].

#### Caries

Dental caries was identified using the WHO criteria as part of the dental health status examination, scoring each dental surface as sound, decayed, missing or filled [29]. They were assessed for each dental surface, evaluating the entire clinical crown including the possibly exposed root. In the fourth simulated screening test, the inter-examiner mean of Kappa values for dental health status among the included cycles was 0.945 (SD: 0.020, range: 0.919–0.981).

For the present analyses, dental caries was expressed at participant-level with measures of extent and prevalence as:

- Untreated dental caries (decayed):
  - Number/proportion of decayed surfaces/teeth (DS/DT);
  - Presence of at least 1 decayed surface/tooth.
- Treated dental caries (filled):
  - Number/proportion of filled surfaces/teeth (FS/FT);
  - Presence of at least 1 filled surface/tooth.
- Cumulative caries experience (decayed and filled):
  - Number/proportion of cumulative caries experience (DFS/DFT scores);
  - Presence of cumulative caries experience (presence of a DFS/DFT score of at least 1).

#### Periodontitis

The periodontal status of the participants was assessed through the Community Periodontal Index (CPI) [29]. The measurements were performed through the walking probing method with a 0.5 mm ball tipped CPI probe. During probing calibration sessions, the examiners were calibrated to apply 20 g probing force approximately. In the fourth simulated screening test, the inter-examiner mean of Kappa values for periodontal status was 0.754 (SD: 0.120, range: 0.518–0.900).

The dentition was split into six sextants represented by the following tooth numbers (FDI system): 18–14, 13–23, 24–28, 38–34, 33–43 and 44–48. A sextant was examined just in case two or more unscheduled teeth for extraction were present. Ten index teeth were used for the examination: #17, #16, #11, #26, #27, #37, #36, #31, #46, #47 (FDI system). If a sextant missed one of the index teeth, an adjacent tooth was examined. If no adjacent tooth was even present, all remaining teeth of that sextant were considered for examination.

The Community Periodontal Index was scored from 0 to 4 as follows: 0 (healthy), 1 (gingival bleeding after probing), 2 (calculus), 3 (PPD between 3.5 and 5.5 mm) and 4 (PPD > 5.5 mm). The score of the sextant was calculated as its highest score.

For the current analysis, the periodontal status at participant-level was dichotomized into:

- “No periodontitis” (CPI ≤ 2 in all sextants);
- “Periodontitis” (CPI ≥ 3 in at least one sextant).

As a sensitivity analysis, a different categorization was also employed, naming it ‘severe periodontitis’, as follows:

- “No severe periodontitis” (CPI ≤ 3 in all sextants);
- “Severe periodontitis” (CPI = 4 in at least one sextant).

#### Covariates

The covariate assessment methods are reported in Appendix S1.

### Statistical analyses

All statistical analyses were carried out with a statistical software (SPSS 23.0 software, IBM Corp, Armonk, NY, USA), using analysis for complex samples with a design-based approach [30]. Furthermore, the stratification, the clustering, and the appropriate sample weights were considered in order to generalize the results to the entire South Korea population. All the reported *p*-values are two-tailed, and the significance was in advance set at *p* < 0.05. All missing data with any assumption were handled with complete case analyses with covariates adjustment, as for current statistic literature orientation (Table S1) [31].

The statistical plan has been elaborated similarly to previous reports [32–35]. Briefly, descriptive characteristics regarding all the covariates were summarized for the whole population and categorized for periodontal status. Categorical data were reported as number (%), while continuous variables were reported as mean (Coefficient of Variation — CV).

In order to evaluate the “crude” (unadjusted) association between periodontitis and caries measurements, the preliminary Odds Ratios (ORs) or Mean Differences (MDs) together with 95% confidence intervals (CIs) were obtained and reported together with the relative *p*-values deriving from an *F*-test.

Multiple linear/logistic regression analyses were then applied to examine the association between periodontitis and each caries measure, adjusting for the potential confounders (selected according to the external knowledge) using the following 7 models:

- *Model 1* (only age and gender);
- *Model 2* (Model 1 + smoking status);
- *Model 3* (Model 2 + frequency of toothbrushing + use of interproximal toothbrush + use of flossing);
- *Model 4* (Model 3 + educational level + household income);
- *Model 5* (Model 4 + gum diseases treatment previous year + tooth filling previous year);
- *Model 6* (Model 5 + BMI + Vitamin D serum levels + AUDIT score + diabetes status + stress);
- *Model 7* (Model 6 + carbohydrates dietary intake).

The ORs/MDs (95% CIs) obtained from the multiple regression analyses were reported, as well as the *p*-values derived from a *F*-test.

## Results

The sampling strategy gave rise to the selection of 50,326 people: 39,964 (79.3%) of them accepted to participate. Of those participants, 37,842 took part to both the health interview and the health examination (75.2% of the total). As previously stated, the final sample weights considered this survey-non-response rate.

The present analysis included only adults (≥ 19 years old) who received the periodontal and caries examination, for a total of 23,405 participants representative of 36.2 million adults (Figure S1).

### Descriptive characteristics of the study population

Table 1 provides descriptive statistics of the study participants, both overall and categorized for periodontal status (for both case definitions—CPI ≥ 3 and CPI = 4). Six thousand nine hundred and sixty-seven participants had periodontitis defined as CPI ≥ 3 (weighted N. 9,627,683 — weighted 26.6%) and one thousand nine hundred and sixty-nine participants had periodontitis defined as CPI = 4 (weighted N. 2,714,136 — weighted 7.5%). The mean number of decayed surfaces was 1.57 (0.026), and six thousand nine hundred and seventy-six participants had at least one decayed tooth (weighted N. 11,478,867 — weighted 31.7%).

**Table 1:** Characteristics of the study population, overall and according to the periodontal status

Variable	Overall*
Age (years), mean (CV)	45.42 (0.004)
Gender, N (%)	
Men	9462 (49.6)
Women	13,943 (50.4)
Smoking Status, N (%)	
Non-smokers	18,564 (76.7)
Current smokers	3983 (23.3)
Tooth brushing frequency, N (%)	
0–1 per day	3482 (14.0)
2 per day	9081 (38.1)
≥ 3 per day	10,842 (47.9)
Use of interproximal toothbrush, N (%)	
No	14,821 (78.8)
Yes	3991 (21.2)
Use of flossing toothbrush, N (%)	
No	14,493 (76.0)
Yes	4319 (24.0)
Educational Level, N (%)	
Primary school	5355 (16.3)
Middle school	2,420 (9.2)
High school	7457 (38.9)
University/College	7003 (35.6)
Monthly household income, N (%)	
Low	4492 (15.0)
Middle low	5911 (25.5)
Middle high	6328 (29.5)
High	6477 (30.0)
Gum diseases treatment last year, N (%)	
No	20,391 (90.9)
Yes	2178 (9.1)
Tooth filling last year, N (%)	
No	19,963 (88.0)
Yes	2606 (12.0)
BMI (kg/m^2^), mean (CV)	23.75 (0.001)
Vitamin D serum levels (ng/dl), mean (CV)	17.02 (0.008)
Alcoholism (AUDIT score), mean (CV)	6.55 (0.011)
Diabetes status, N (%)	
Normal	14,145 (71.6)
Impaired Fasting Glucose (IFG)	4311 (19.8)
Diabetes	2191 (8.5)
Stress, N (%)	
No/slightly stressed	16,913 (73.4)
Moderately/highly stressed	5628 (26.6)
Carbohydrates intake (g), mean (CV)	321.36 (0.004)

Data are presented as unweighted N (weighted %) for binary and categorical variables and as weighted mean (CV) for continuous variables

N is the unweighted population size for each group; the sum of N varied according to missing data for each variable

^*^Overall: N(unweighted) = 23,405; n(weighted) = 36,179,177

^†^CPI ≥ 3 case definition:

- No periodontitis: N(unweighted) = 16,438; n(weighted) = 26,551,495;

- Periodontitis: N(unweighted) = 6967; n(weighted) = 9627,683

^‡^ CPI = 4 case definition:

- No Severe Periodontitis: N(unweighted) = 21,436; n(weighted) = 33,465,041;

- Severe Periodontitis: N(unweighted) = 1969; n(weighted) = 2,714,136

### Periodontitis and untreated dental caries (decayed)

In the unadjusted models, the presence of periodontitis (both case definitions) was directly associated with presence/number of decayed teeth/surfaces.

After controlling for the selected confounders, a significant direct association continued to be present in almost all models. In model 7, participants with periodontitis (CPI ≥ 3) had a mean of 0.82 (95% CI: 0.41–1.23) more decayed surfaces and of 0.36 (95% CI: 0.22–0.50) more decayed teeth than participants without periodontitis, with an OR to have at least one decayed surface/tooth of 1.96 (95% CI: 1.66–2.32) (Table 2). The estimates for severe periodontitis were lower, but mostly still significant (Table 3).

**Table 2:** Adjusted MDs (95% CIs) and ORs (95% CIs) for different caries measurements (untreated, treated and cumulative caries experience) according to the prevalence of periodontitis (CPI ≥ 3 case definition)

Variables	Crude model (Mean 95% CI)	Model 1 (Mean 95% CI)	Model 2 (Mean 95% CI)	Model 3 (Mean 95% CI)	Model 4 (Mean 95% CI)	Model 5 (Mean 95% CI)	Model 6 (Mean 95% CI)	Model 7 (Mean 95% CI)
Untreated dental caries (decayed)								
Number of decayed surfaces								
* MD (95% CI)*	0.85‡ (0.67/1.02)	0.65‡ (0.45/0.86)	0.63‡ (0.43/0.84)	0.53‡ (0.31/0.75)	0.49‡ (0.29/0.70)	0.52‡ (0.32/0.73)	0.83‡ (0.42/1.24)	0.82‡ (0.41/1.23)
Percentage of decayed surfaces								
* MD (95% CI)*	1.15‡ (0.87/1.43)	0.26 (− 0.10/0.63)	0.39* (0.07–0.72)	0.24 (− 0.13/0.60)	0.23 (− 0.08/0.53)	0.27 (− 0.05/0.58)	0.91‡ (0.40/1.41)	0.91‡ (0.40/1.41)
Number of decayed teeth								
* MD (95% CI)*	0.21‡ (0.15/0.27)	0.28‡ (0.21/0.34)	0.26‡ (0.19/0.33)	0.23‡ (0.14/0.30)	0.21‡ (0.14/0.29)	0.23‡ (0.15/0.30)	0.36‡ (0.22/0.50)	0.36‡ (0.22/0.50)
Percentage of decayed teeth								
* MD (95% CI)*	1.49‡ (1.11/1.87)	0.89‡ (0.46/1.32)	0.97‡ (0.57/1.37)	0.75‡ (0.30/1.19)	0.72‡ (0.33/1.12)	0.78‡ (0.38/1.19)	1.66‡ (1.01/2.32)	1.66‡ (1.01/2.32)
Presence of at least 1 decayed surface/tooth—OR (95% CI)								
* No*	*Ref*	*Ref*	*Ref*	*Ref*	*Ref*	*Ref*	*Ref*	*Ref*
* Yes*	1.40‡ (1.30/1.52)	1.54**‡** (1.42/1.68)	1.50‡ (1.38/1.63)	1.48‡ (1.35/1.62)	1.47**‡** (1.34/1.62)	1.51**‡** (1.38/1.66)	1.97**‡** (1.66/2.32)	1.96**‡** (1.66/2.32)
Treated dental caries (filled)								
Number of filled surfaces								
* MD (95% CI)*	− 1.36‡ (− 1.80/ − 0.93)	− 1.08**‡** (− 1.53/ − 0.63)	− 1.10‡ (− 1.46/ − 0.54)	− 0.69† (− 1.18/ − 0.19)	− 0.49 (− 0.99/ − 0.01)	− 0.50 (− 1.00/0.01)	− 0.65 (− 1.57/0.27)	− 0.66 (− 1.58/0.27)
Percentage of filled surfaces								
* MD (95% CI)*	− 0.24 (− 0.65/0.17)	− 0.99‡ (− 1.41/ − 0.56)	− 0.96‡ (− 1.39/ − 0.52)	− 0.78‡ (− 1.26/ − 0.31)	− 0.62* (− 1.11/ − 0.14)	− 0.63* (− 1.11/ − 0.15)	− 0.68 (− 1.52/0.16)	− 0.69 (− 1.53/0.15)
Number of filled teeth								
* MD (95% CI)*	− 1.35‡ (− 1.49/ − 1.22)	− 0.57**‡** (− 0.71/ − 0.44)	− 0.52‡ (− 0.66/ − 0.38)	− 0.40‡ (− 0.55/ − 0.25)	− 0.33**‡** (− 0.48/ − 0.18)	− 0.32**‡** (− 0.47/ − 0.17)	− 0.46† (− 0.72/ − 0.20)	− 0.46† (− 0.72/ − 0.20)
Percentage of filled teeth								
* MD (95% CI)*	− 3.89‡ (− 4.42/ − 3.36)	− 2.10‡ (− 2.64/ − 1.56)	− 1.96‡ (− 2.52/ − 1.41)	− 1.58‡ (− 2.18/ − 0.98)	− 1.33‡ (− 1.93/ − 0.73)	− 1.32‡ (− 1.91/-0.73)	− 1.74‡ (− 2.80/ − 0.73)	− 1.74‡ (− 2.80/ − 0.73)
Presence of at least 1 filled surface/tooth—OR (95% CI)								
* No*	*Ref*	*Ref*	*Ref*	*Ref*	*Ref*	*Ref*	*Ref*	*Ref*
* Yes*	0.59‡ (0.55/0.64)	0.77**‡** (0.70/0.84)	0.78‡ (0.72/0.86)	0.82‡ (0.74/0.91)	0.84† (0.76/0.93)	0.83† (0.75/0.92)	0.73† (0.60/0.88)	0.73† (0.60/0.88)
Cumulative caries experience (decayed and filled)								
DFS score								
* MD (95% CI)*	− 0.52* (− 0.96/ − 0.07)	− 0.43 (− 0.90/0.04)	− 0.36 (− 0.84/0.12)	− 0.16 (− 0.68/0.37)	0.00 (− 0.53/0.53)	0.03 (− 0.51/0.56)	0.17 (− 0.87/1.21)	0.17 (− 0.88/1.21)
Percentage of decayed/filled surfaces								
* MD (95% CI)*	0.91‡ (0.42/1.40)	− 0.72* (− 1.28/-0.17)	− 0.56* (− 1.09/ − 0.03)	− 0.55 (− 1.11/0.04)	− 0.40 (− 0.97/0.18)	− 0.36 (− 0.94/0.21)	0.22 (− 0.80/1.24)	0.22 (− 0.80/1.24)
DFS/DFT score prevalences—OR (95% CI)								
* DFS/DFT score* = *0*	*Ref*	*Ref*	*Ref*	*Ref*	*Ref*	*Ref*	*Ref*	*Ref*
* DFS/DFT score* ≥ *1*	0.73**‡** (0.66/0.80)	0.98 (0.88/1.09)	0.98 (0.88/1.09)	1.04 (0.92/1.17)	1.06 (0.93/1.20)	1.06 (0.94/1.21)	1.02 (0.79/1.31)	1.02 (0.79/1.31)
DFT score								
* MD (95% CI)*	− 1.14**‡** (− 1.28/ − 1.00)	− 0.30**‡** (− 0.43/ − 0.16)	− 0.26‡ (− 0.40/ − 0.12)	− 0.17* (− 0.33/ − 0.012)	− 0.11 (− 0.27/0.05)	− 0.10 (− 0.25/0.06)	− 0.10 (− 0.40/0.19)	− 0.11 (− 0.40/0.19)
Percentage of decayed/filled teeth								
* MD (95% CI)*	− 2.40‡ (− 3.01/ − 1.79)	− 1.21‡ (− 1.87/ − 0.56)	− 1.00† (− 1.63/ − 0.36)	− 0.84* (− 1.53/ − 0.14)	− 0.61 (− 1.29/0.08)	− 0.54 (− 1.22/0.15)	− 0.08 (− 1.27/1.10)	− 0.08 (− 1.27/1.11)

^*^Statistically significant (*p* < 0.05)

^†^Statistically significant (*p* < 0.01);

^**‡**^ Statistically significant (*p* < 0.001)

The independent variable is periodontitis and the dependent variable is a caries measurement

Model 1: adjusted for age and gender

Model 2: variables in Model 1 plus smoking status

Model 3: variables in Model 2 plus frequency of toothbrushing, use of interproximal toothbrush and use of flossing

Model 4: variables in Model 3 plus educational level and income

Model 5: variables in Model 4 plus gum diseases treatment last year and tooth filling last year

Model 6: variables in Model 5 plus BMI, Vitamin D levels, AUDIT score, diabetes mellitus, and stress

Model 7: variables in Model 6 plus carbohydrates intake

**Table 3:** Adjusted MDs (95% CIs) and ORs (95% CIs) for different caries measurements (untreated, treated and cumulative caries experience) according to the prevalence of severe periodontitis (CPI = 4 case definition)

Variables	Crude model (Mean 95% CI)	Model 1 (Mean 95% CI)	Model 2 (Mean 95% CI)	Model 3 (Mean 95% CI)	Model 4 (Mean 95% CI)	Model 5 (Mean 95% CI)	Model 6 (Mean 95% CI)	Model 7 (Mean 95% CI)
Untreated dental caries (decayed)								
Number of decayed surfaces								
* MD (95% CI)*	0.71‡ (0.43/0.99)	0.45† (0.16/0.74)	0.41† (0.11/0.72)	0.31 (− 0.01/0.62)	0.25 (− 0.04/0.55)	0.29 (− 0.00/0.59)	0.32 (− 0.14/0.79)	0.33 (− 0.14/0.79)
Percentage of decayed surfaces								
* MD (95% CI)*	0.97‡ (0.57/1.36)	0.06 (− 0.04/0.51)	0.14 (− 0.30/0.58)	− 0.08 (− 0.57/0.40)	− 0.08 (− 0.52/0.36)	− 0.03 (− 0.47/0.41)	0.19 (− 0.44/0.81)	0.19 (− 0.44/0.81)
Number of decayed teeth								
* MD (95% CI)*	0.17† (0.07/0.27)	0.20‡ (0.10/0.31)	0.19‡ (0.08/0.30)	0.13* (0.02/0.24)	0.11* (0.01/0.21)	0.12* (0.02/0.23)	0.19* (0.01/0.37)	0.19* (0.01/0.37)
Percentage of decayed teeth								
* MD (95% CI)*	1.22‡ (0.67/1.77)	0.53 (− 0.07/1.13)	0.57 (− 0.04/1.18)	0.22 (− 0.43/0.88)	0.18 (− 0.41/0.78)	0.26 (− 0.34/0.85)	0.97* (0.02/1.91)	0.96* (0.02/1.91)
Presence of at least 1 decayed surface/tooth—OR (95% CI)								
* No*	*Ref*	*Ref*	*Ref*	*Ref*	*Ref*	*Ref*	*Ref*	*Ref*
* Yes*	1.37‡ (1.21/1.54)	1.44‡ (1.27/1.62)	1.39‡ (1.22/1.58)	1.34‡ (1.16/1.55)	1.32‡ (1.14/1.52)	1.36‡ (1.18/1.57)	1.78‡ (1.32/2.39)	1.78‡ (1.32/2.39)
Treated dental caries (filled)								
Number of filled surfaces								
* MD (95% CI)*	− 2.10‡ (− 2.73/ − 1.47)	− 1.70‡ (− 2.33/ − 1.06)	− 1.53‡ (− 2.20/ − 0.87)	− 1.04† (− 1.77/ − 0.32)	− 0.95* (− 1.68/ − 0.22)	− 0.95* (− 1.70/ − 0.21)	− 0.60 (− 2.12/0.93)	− 0.60 (− 2.12/0.93)
Percentage of filled surfaces								
* MD (95% CI)*	− 0.79* (− 1.43/ − 0.16)	− 1.39‡ (− 2.03/ − 0.76)	− 1.31‡ (− 1.97/ − 0.65)	− 1.02† (− 1.75/ − 0.29)	− 0.94* (− 1.68/ − 0.19)	− 0.94* (− 1.70/ − 0.18)	− 0.48 (− 1.98/1.01)	− 0.48 (− 1.98/1.02)
Number of filled teeth								
* MD (95% CI)*	− 1.46‡ (− 1.65/ − 1.27)	− 0.63‡ (− 0.82/ − 0.44)	− 0.56‡ (− 0.76/ − 0.36)	− 0.40‡ (− 0.62/ − 0.19)	− 0.37† (− 0.58/ − 0.16)	− 0.36† (− 0.57/ − 0.14)	− 0.38 (− 0.79/0.04)	− 0.37 (− 0.79/0.04)
Percentage of filled teeth								
* MD (95% CI)*	− 4.21‡ (− 4.99/ − 3.43)	− 2.20‡ (− 2.98/ − 1.41)	− 2.00‡ (− 2.82/ − 1.18))	− 1.52‡ (− 2.42/ − 0.63)	− 1.38† (− 2.28/ − 0.49)	− 1.36† (− 2.27/ − 0.45)	− 1.25 (− 2.97/0.48)	− 1.24 (− 2.97/0.49)
Presence of at least 1 filled surface/tooth—OR (95% CI)								
* No*	*Ref*	*Ref*	*Ref*	*Ref*	*Ref*	*Ref*	*Ref*	*Ref*
* Yes*	0.56‡ (0.50/0.63)	0.74‡ (0.65/0.83)	0.74‡ (0.65/0.85)	0.84* (0.72/0.97)	0.84* (0.73/0.98)	0.83* (0.72/0.97)	0.81 (0.60/1.10)	0.81 (0.60/1.10)
Cumulative caries experience (decayed and filled)								
DFS score								
* MD (95% CI)*	− 1.39‡ (− 2.07/-0.72)	− 1.25‡ (− 1.93/ − 0.57)	− 1.12† (− 1.83/ − 0.41)	− 0.74 (− 1.51/0.03)	− 0.70 (− 1.47/0.08)	− 0.66 (− 1.45/0.13)	− 0.28 (− 1.83/1.28)	− 0.27 (− 1.83/1.29)
Percentage of decayed/filled surfaces								
* MD (95% CI)*	0.17 (− 0.58/0.92)	− 1.34† (− 2.12/ − 0.56)	− 1.18† (− 1.95/ − 0.40)	− 1.10* (− 1.96/ − 0.25)	− 1.02* (− 1.87/ − 0.16)	− 0.97* (− 1.84/ − 0.10)	− 0.30 (− 1.83/1.23)	− 0.29 (− 1.83/1.24)
DFS/DFT score prevalences—OR (95% CI)								
* DFS/DFT score* = *0*	*Ref*	*Ref*	*Ref*	*Ref*	*Ref*	*Ref*	*Ref*	*Ref*
* DFS/DFT score* ≥ *1*	0.70‡ (0.61/0.81)	0.94 (0.81/1.09)	0.95 (0.81/1.11)	1.06 (0.89/1.27)	1.07 (0.89/1.28)	1.08 (0.90/1.29)	1.16 (0.77/1.74)	1.16 (0.78/1.74)
DFT score								
* MD (95% CI)*	− 1.29‡ (− 1.50/ − 1.09)	− 0.43‡ (− 0.63/ − 0.22)	− 0.37† (− 0.60/ − 0.16)	− 0.27* (− 0.50/ − 0.04)	− 0.26* (− 0.48/ − 0.03)	− 0.23* (− 0.46/ − 0.00)	− 0.19 (− 0.62/0.25)	− 0.19 (− 0.62/0.25)
Percentage of decayed/filled teeth								
* MD (95% CI)*	− 2.99‡ (− 3.92/ − 2.07)	− 1.67† (− 2.63/ − 0.71)	− 1.43† (− 2.42/ − 0.44)	− 1.30† (− 2.37/-0.24)	− 1.20† (− 2.24/-0.16)	− 1.11* (− 2.16/-0.06)	− 0.28 (− 2.08/1.52)	− 0.28 (− 2.08/1.52)

^*^Statistically significant (*p* < 0.05)

^†^Statistically significant (*p* < 0.01)

^‡^Statistically significant (*p* < 0.001)

The independent variable is severe periodontitis and the dependent variable is a caries measurement

Model 1: adjusted for age and gender

Model 2: variables in Model 1 plus smoking status

Model 3: variables in Model 2 plus frequency of toothbrushing, use of interproximal toothbrush and use of flossing

Model 4: variables in Model 3 plus educational level and income

Model 5: variables in Model 4 plus gum diseases treatment last year and tooth filling last year

Model 6: variables in Model 5 plus BMI, Vitamin D levels, AUDIT score, diabetes mellitus, and stress

Model 7: variables in Model 6 plus carbohydrates intake

### Periodontitis and treated dental caries (filled)

In the crude models, periodontitis (both case definitions) was inversely associated with the presence/number of filled teeth/surfaces. In the multiple regression models the association — when present — was always still inverse. In particular, in Model 7 participants with periodontitis (CPI ≥ 3) had an OR of 0.73 (95% CI: 0.60–0.88) to have at least one filled surface/tooth, while the estimate was not significant for severe periodontitis (OR = 0.81; 95% CI: 0.60–1.10).

### Periodontitis and cumulative caries experience (decayed and filled)

In the univariate models, cumulative caries experience was inversely associated with both periodontitis case definitions, with the only exception of percentage of DFS with severe periodontitis.

After controlling for possible confounders, cumulative caries experience and periodontitis (both case definitions) were not associated in all the multiple regression models (Tables 2 and 3).

## Discussion

In this study, periodontitis has shown to be independently associated with an increase in the number of untreated decayed teeth and with a decrease in the number of filled teeth. In particular, the subjects with periodontitis had an increase of 96% in the estimated odds to have at least one untreated caries, while they had reduced estimated odds to have at least one filled tooth. Conversely, periodontitis was not associated with cumulative caries experience after adjusting for known common risk factors. Sensitivity analyses for severe periodontitis showed mostly consistent results, however the estimates were generally lower than for CPI ≥ 3 case definition.

Untreated and treated dental caries demonstrated divergent associations with periodontitis in our cohort. This finding is likely to be explained by a possible residual confounding by access to dental care. Indeed, we defined periodontitis based on PPD (CPI index), which is modifiable in nature by periodontal treatment, as dental caries status can be modified by fillings. When looking however at cumulative caries experience, a higher prevalence of both decayed and filled teeth could have also been expected in individuals with periodontitis. Indeed, clinical attachment loss due to periodontal destruction may result in an increased tooth surface area exposed to the oral cavity [36, 37], which confer an intrinsic increased risk for root caries development [38, 39]. However, cumulative caries experience and periodontitis were not associated in our cohort. This finding can be again interpreted in light of the use of PPD to define periodontitis instead of CAL, as well as to the heterogeneous characteristics of supra- and subgingival plaque. Indeed, root surface exposure within a subgingival microenvironment is more likely colonized by an anaerobic proteolityc biofilm than from an acidogenic flora characteristic of dental caries.

Results of investigations on a simultaneous experience of caries and periodontitis have not been consistent so far. On the one hand, authors of the initial quantitative studies comparing the occurrence of cavities and calculus proposed the concept of an inverse relationship between caries and periodontitis [40]. However, several studies were not able to confirm this initial hypothesis, either stating an inaccuracy in supporting an inverse theory [41] or highlighting no association between cumulative caries experience and periodontitis [21, 42, 43]. Similarly to our findings, Albandar et al. highlighted a significant association between the presence of cavities, non-defective and defective fillings, and the progression of periodontitis [20]. Moreover, Mattila et al. in 2010 confirmed again that severe periodontitis and untreated dental caries tended to co-occur in the same subjects [23].

The results from this study should be regarded with caution due the above-mentioned risk of residual confounding (e.g., supragingival plaque, gingival recession) and due to its cross-sectional design, which prevent from any causal inferences. Moreover, a risk of information bias may exist in relation to the assessment of both periodontal status and dental caries. Indeed, periodontitis in KNHANES was assessed by the use of CPI, which is a partial mouth examination protocol only based on PPD [44–46]. Similarly, the caries assessment method applied in KNHANES may underestimate the presence and the extent of the disease, since early, non-cavitated lesions could not be detected [47], and radiographic examination was not performed in order to evaluate the involvement of approximal surfaces [48, 49]. Moreover, it does not allow to stage severity of caries and does not differentiate between active and inactive lesions [47]. Finally, part of the missing data might be missing-not-at-random, as they could be lacking due to edentulism caused by either periodontitis or caries. This could potentially have an impact on our results, as edentulous participants could have both periodontitis and dental caries, but they were systematically missing from our data because they had possibly lost their teeth as a result of both diseases. Nonetheless, this study possesses the novelty to having analysed the co-occurrence of treated and untreated dental caries through multivariate analyses. Its external validity is also maximized by the employed sampling procedures, which allow the generalizability of the present findings to the whole non-institutionalized South-Korean population.

The results presented here are advisable for further investigation. These findings need to be replicated in other populations, possibly employing a full-mouth periodontal examination protocol to allow a site-level analysis, and verifying if the results may change depending on whether clinical attachment loss affects the supra-(recession) or sub-(increased PPD) gingival environment. Moreover, the information on dental caries should be completed through radiographs, measures of severity and distinction among coronal and radicular caries [48, 50]. Researchers should take countermeasures to minimize the risk of residual confounding, particularly by collecting detailed data about exposure to periodontal and dental care. Longitudinal and mechanistic studies are also needed.

## Conclusions

In this large nationally representative population, treated and untreated dental caries demonstrated divergent associations with periodontitis. However, when considering cumulative caries experience, the two diseases appeared not to be related.

In light of their possible co-occurrence, clinicians should implement integrative diagnostic, preventive and treatment strategies for both diseases.

### Supplementary Information

Below is the link to the electronic supplementary material.

Supplementary file1 (DOCX 17 KB)

Supplementary file2 (TIFF 1299 KB)

Supplementary file3 (DOCX 17 KB)

## Data Availability

The data that support the findings of this study are listed in the main manuscript and supporting material.
