# Supplementary material for: The co-occurrence of the two main oral diseases: periodontitis and dental caries
Source: Clin Oral Investig. 2023 Sep 16;27(11):6483–92. doi: 10.1007/s00784-023-05253-2 (PMC10630193; doi:10.1007/s00784-023-05253-2)
Supplement: Supplementary file 1 — Supplementary file1 (DOCX 17 KB) [file 784_2023_5253_MOESM1_ESM.docx]

**Appendix S1.** Covariate assessment methods.

As socio-demographic characteristics, the following variables were considered: age, gender, educational level and monthly household income. The educational level was evaluated as the highest diploma obtained (primary school, middle school, high school and university/college). Monthly household income was assessed as the family income adjusted for the number of family members; on this basis, participants were classified, in the initial sample, into quartiles groups (low, middle low, middle high and high).

As systemic health variables, Body Mass Index (BMI), diabetes status and serum levels of vitamin D were considered. All of them were measured during the health examination. Body height and weight were respectively measured to the nearest 0.1 cm and 0.1 kg, with the participants in light indoor clothing without shoes. BMI was then calculated (kg/m²). Fasting plasma glucose (FPG) was measured by an enzymatic method using a Hitachi automatic analyzer 7600 (Hitachi, Tokyo, Japan). Diabetes status was then categorized as non-diabetic (FPG<100mg/dL), impaired fasting glycaemia - IFG (100≤FPG<126mg/dL) or diabetic (FPG≥126mg/dL or medicated for diabetes). Serum level of Vitamin D [25(OH)D] was measured with a radioimmunoassay (RIA) kit (DiaSorin Inc., Stillwater, USA) using a gamma-counter (1470Wizard; PerkinElmer, Turku, Finland).

As general health behaviors, the following self-reported variables were considered: smoking status, alcoholism and stress. Regarding smoking status, the subjects were divided into two groups: non-smokers (including former smokers) and current smokers. The Alcohol Use Disorders Identification Test (AUDIT) score (0 to 40) was used to evaluate alcoholism, as per guidance of WHO.^1^ Self-reported stress was categorized as no/slightly stressed and moderately/highly stressed.

As self-reported oral health behaviors, the toothbrushing frequency per day (0-1 time/day, 2 times/day, ≥3 times/day) and the use or not of dental floss and interproximal toothbrush were considered. Moreover, self-reported gum diseases treatment and tooth filling (no/yes) in the previous year were collected.

As nutritional variable, the dietary intake of carbohydrates was considered. It was assessed with the 24-h recall method by trained dietitians, using the reference Food Composition Table.^2^

**Appendix S1 references:**

1. Babor TF, Higgins-Biddle JC, Saunders JBJ, Monteiro M.G. AUDIT, the Alcohol Use Disorder Identification Test – 2nd edition. Geneva: World Health Organization; 2001.
2. National Rural Resources Development Institute. Food Composition Table - 7th edition. Suwon: Rural Development Administration; 2006.
