# Supplementary figures and images for: The co-occurrence of the two main oral diseases: periodontitis and dental caries
Source: Clin Oral Investig. 2023 Sep 16;27(11):6483–92. doi: 10.1007/s00784-023-05253-2 (PMC10630193; doi:10.1007/s00784-023-05253-2)

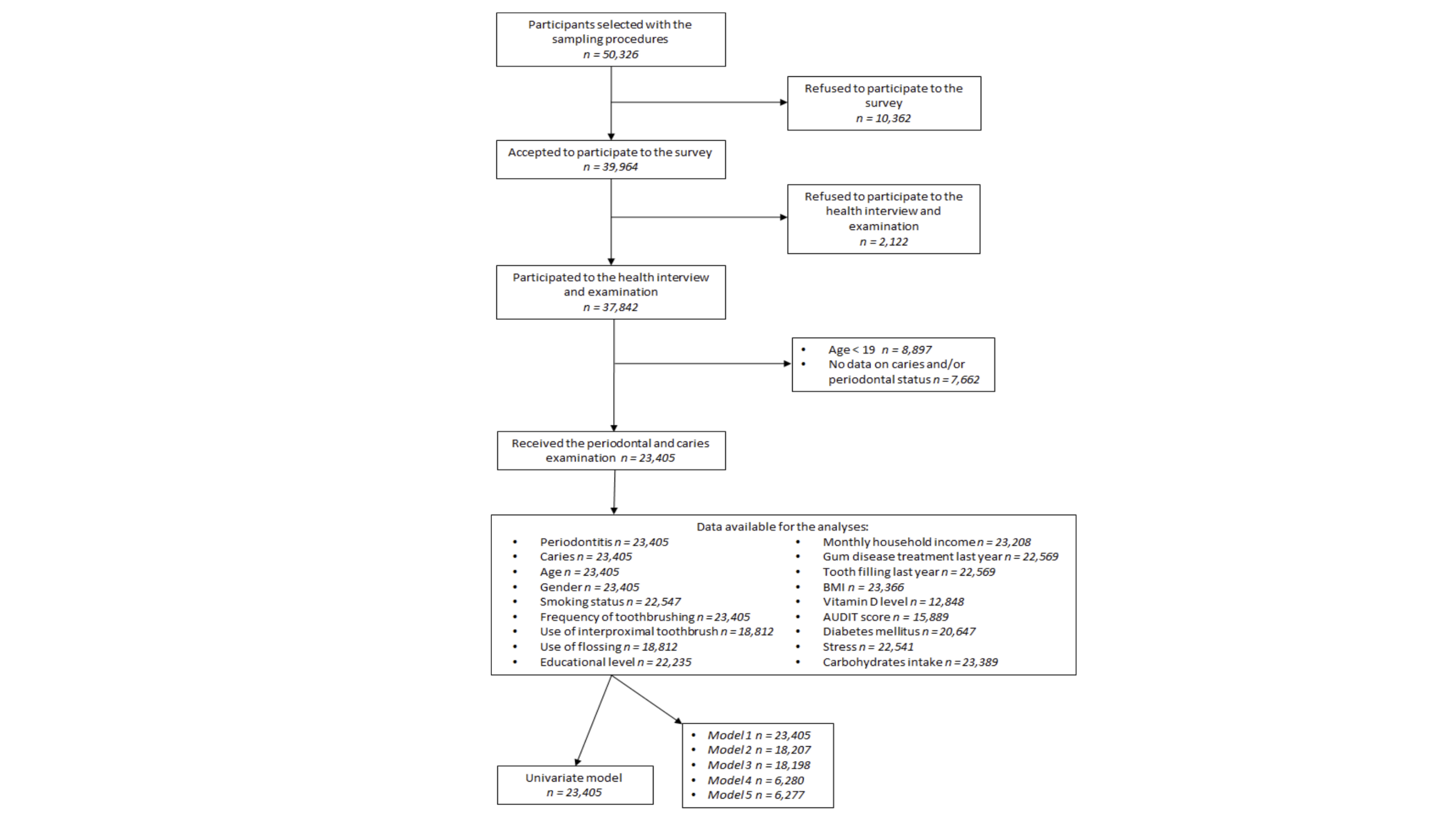

Supplement: Supplementary file 2 — Supplementary file2 (TIFF 1299 KB) [file 784_2023_5253_MOESM2_ESM.tiff]
