# Supplementary material for: The co-occurrence of the two main oral diseases: periodontitis and dental caries
Source: Clin Oral Investig. 2023 Sep 16;27(11):6483–92. doi: 10.1007/s00784-023-05253-2 (PMC10630193; doi:10.1007/s00784-023-05253-2)
Supplement: Supplementary file 3 — Supplementary file3 (DOCX 17 KB) [file 784_2023_5253_MOESM3_ESM.docx]

**Table S1.** Missing data

Amount of missing data in the study population (n=23,405) for each variable considered. Missing data were handled with complete case analyses with covariates adjustment [1].

| **Variable** | **Collected data, N (%)** | **Missing data, N (%)** |
| --- | --- | --- |
| Periodontitis | 23,405 (100.0) | 0 (0.0) |
| Tooth status (caries) information | 23,405 (100.0) | 0 (0.0) |
| The number of teeth | 23,405 (100.0) | 0 (0.0) |
| Age | 23,405 (100.0) | 0 (0.0) |
| Gender | 23,405 (100.0) | 0 (0.0) |
| Smoking Status | 22,547 (96.3) | 858 (96.3) |
| Tooth brushing frequency | 23,405 (100.0) | 0 (0.0) |
| Use of interproximal toothbrush | 18,812 (80.4) | 4,593 (80.4) |
| Use of flossing | 18,812 (80.4) | 4,593 (80.4) |
| Educational Level | 22,235 (95.0) | 1,170 (95.0) |
| Monthly household income | 23,208 (99.2) | 197 (99.2) |
| Gum diseases treatment last year | 22,569 (96.4) | 836 (96.4) |
| Tooth filling last year | 22,569 (96.4) | 836 (96.4) |
| BMI | 23,366 (99.8) | 39 (99.8) |
| Vitamin D serum levels | 12,848 (54.9) | 10,557 (54.9) |
| Alcoholism | 15,889 (67.9) | 7,516 (67.9) |
| Diabetes status | 20,647 (88.2) | 2,758 (88.2) |
| Stress | 22,541 (96.3) | 864 (96.3) |
| Carbohydrates intake | 23,389 (99.9) | 16 (99.9) |

**Table S1 references**

1. Groenwold RH, Donders AR, Roes KC, et al (2012) Dealing with missing outcome data in randomized trials and observational studies. American journal of epidemiology 175:210–7. https://doi.org/10.1093/aje/kwr302
